# Selective selfishness in alarm calling behaviour by some members of wintering mixed-species groups of crested tits and willow tits

**DOI:** 10.1098/rstb.2022.0102

**Published:** 2023-06-05

**Authors:** Tatjana Krama, Ronalds Krams, Didzis Elferts, Kathryn E. Sieving, Indrikis A. Krams

**Affiliations:** ^1^ Department of Biotechnology, Daugavpils University, Daugavpils, 5404 Latvia; ^2^ Chair of Plant Health, Estonian University of Life Sciences, Tartu 51006, Estonia; ^3^ Department of Botany and Ecology, Faculty of Biology, University of Latvia, Riga 1004, Latvia; ^4^ Department of Zoology and Animal Ecology, Faculty of Biology, University of Latvia, Riga 1004, Latvia; ^5^ Department of Wildlife Ecology and Conservation, University of Florida, Gainesville, FL 32611, USA; ^6^ Institute of Ecology and Earth Sciences, University of Tartu, Tartu 50409, Estonia

**Keywords:** communication, mixed-species groups, parids, alarm calls, dominance hierarchies, survival

## Abstract

Animals adjust their use of alarm calls depending on social environments. We tested whether dominant (adult) and subordinate (juvenile non-kin) male crested tits (*Lophophanes cristatus*) warn each other and heterospecific willow tits (*Poecile montanus*) across the wintering season. Birds rarely alarm called when feeding alone. Both adult and juvenile crested tits warned each other in early winter, and adults did so in the middle of wintering season. However, juvenile males rarely warned conspecific adult males in the middle of the winter. Both adult and juvenile males stopped giving alarm calls when feeding together at the end of wintering season. The results suggest that the mid-winter reduction of juvenile alarms could increase the likelihood of successful predator attacks on adults, increasing the chances for juveniles to replace adults and acquire their territories. By contrast, both adult and juvenile males produced alarm calls throughout the season when foraging together with willow tits. Whether juvenile male crested tits could be selectively altering alarm call propensity to endanger adult males, thereby selfishly enhancing their own succession to territory ownership, is discussed. The results add to the understanding of the origin of mixed-species groups and explain the dynamics of social communication.

This article is part of the theme issue ‘Mixed-species groups and aggregations: shaping ecological and behavioural patterns and processes’.

## 1. Introduction

### (a) Costs and benefits of living in groups

A major advantage of group living in animals is ready access to the localized concentration of information concerning risks and resources conveyed in the vocalizations, and other modalities of signals and cues, produced by group members [1]. Birds in the Holarctic family Paridae (parids) regularly participate in mixed-species wintering flocks, comprised a variety of species and juveniles and adults of both sexes. In addition to the benefits of group living, the costs of coping with inter- and especially intra-specific aggression in mixed-species groups are well documented in parid flocks [2–5]. Both benefits and costs can accrue differently to individuals in these complex social groups depending on a variety of individual traits (including species, sex, age and dominance status) and environmental conditions.

### (b) Social organization of parids

The Paridae family (chickadees, tits and titmice) includes bird species possessing complex social structures and rich vocal repertoires. In the non-breeding season, many parid species are social, they live in dominance-structured groups and store food in a scattered distribution within their territories. These groups are territorial, stable in space and time and often contain several other parid species [1,2]. The birds jointly defend their territory from neighbouring multi-species groups. Dominance hierarchies are usually stable and arise between conspecific and heterospecific group members [2,3]. Social status affects the survival prospects of individual parids owing to differential access to food resources and exposure to predators in different parts of the tree canopy. Many studies showed that winter mortality of dominants is lower than that of subordinate individuals [4–6].

During the breeding season, European crested tits (*Lophophanes cristatus*) defend breeding territories and raise their offspring. Juvenile crested tits disperse from their natal territories after gaining independence, while their parents are joined by other non-kin, juvenile conspecifics with whom they form winter groups. Typical dispersal distances of food-hoarding parids are only 1–3 territories from their natal territories [7]. Importantly, the process of choosing and becoming permanent winter group members may take four to six weeks, suggesting severe competition for the vacancies available in prospective winter groups. It is also likely that acceptance into a breeding pair's group may be determined by particular juvenile traits such as personality type, that benefit adults' survival.

Predation has been recognized as the most important factor responsible for the mortality of parids wintering at high latitudes [5,8]. Socially connected individuals often detect an attacking predator significantly sooner than solitary parids, which improves social foraging in tit winter groups via shared vigilance. Parids and many other bird species utter specific aerial alarm calls to warn conspecific individuals when they detect approaching hawks or falcons [9]. Thus, successful predator avoidance and improved survival are possible if only group members honestly warn each other. Therefore, a relatively long trial period for a juvenile seeking membership in a winter group allows the juvenile to assess the pair of adults’ willingness to warn juveniles and, in turn, adults can determine the juvenile's warning behaviours under dangerous situations.

The benefits of being social often differ between dominant individuals (e.g. older adults) and subordinates (e.g. inexperienced juveniles). While adults and juveniles clearly benefit from the presence of each other via risk dilution and the surprise effect, juveniles could accrue the benefits of territory inheritance by avoiding warning their dominant group mates and enhancing adult mortality [2,7,8]. This is hypothesized to be most likely in social systems where new dominants in groups are recruited from the bottom of the rank order [10], and high-rank positions become available as the present dominant group members die [7]. Therefore, juvenile birds joining a group may avoid giving alarm calls when an approaching predator is detected because this can increase the likelihood of the predator killing the dominants. As winter ends and birds begin breeding cycles, dominants must expel juveniles. Since territory ownership is the only pathway for juvenile crested tits to breed in an upcoming reproductive season, attempts to conceal predator approaches from dominants may be especially likely during the aggression-laden break-up of winter social flocks.

### (c) Parid alarm calls and social status

**Figure 1.**
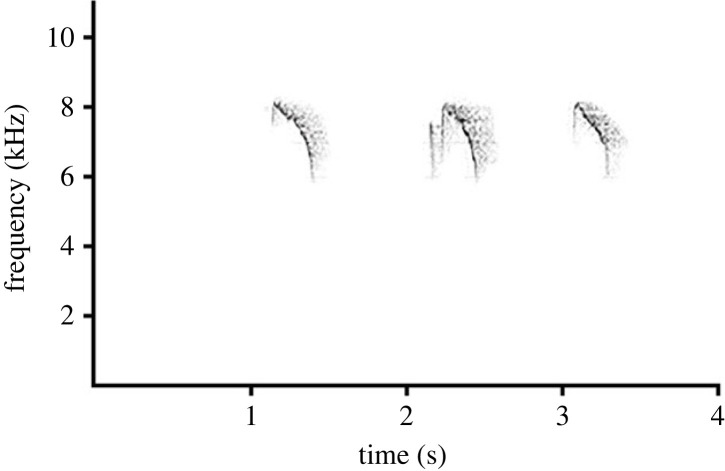
Aerial alarm calls of crested tits.

Marler [11] showed that ‘seeet’ aerial alarm calls of many European birds are high-pitched (6–10 kHz) and this structure is similar across many European bird species and highly conserved within the family Paridae across its Holarctic range [12]. The high-pitch and narrow frequency modulation of such calls makes them, both, difficult for an immediate localization of the caller by predators [13], and readily recognizable to nearby prey across many different types of species [14,15]. Crested tits produce such a ‘seeet’ alarm call when detecting airborne predators such as hawks (figure 1) [16]. This call is also used fraudulently at times as a false alarm call. Subdominant great tits may give false alarm calls in the presence of their more dominant group mates to access food sources at feeders [17]. Birds hearing false alarm calls usually dive to cover and so giving false alarm calls to reduce competition is a highly manipulative behaviour suggesting sophisticated cognition [18]. While fraudulent alarm calling supposedly does not affect the survival of signal receivers, avoiding warning dominant group mates could reflect an adaptation to enhance winter mortality risk for a dominant conspecific competitor by its subordinate rival [19,20].

Understanding how the value of social communication varies under the context of anti-predator protection across dominance hierarchies in mixed-species parid groups is relevant to ecology and the behavioural sciences [21]. To test whether juvenile males withhold alarm calls when in the company of only adult males of their winter social groups, we conducted a two-step experiment. First, we caught adult and juvenile crested tits in a group without focal individuals being aware to create certain social situations and then simulated a hawk attack by gliding a small model of a sparrowhawk towards males feeding at feeders in the forest. The model was pretested to determine that it usually elicits focal crested tits to give aerial alarm calls. Following presentations of the glider, we observed the alarm call-giving behaviour of adult and juvenile crested tit males. We predicted that adult crested tit males would warn juvenile crested tit males at the beginning and the middle of the non-reproductive season while they may be less willing to give alarm calls to warn juvenile males at the end of the non-breeding season. We also predicted that juvenile crested tit males would warn adult males at the beginning of the winter season. We expected that juvenile males would avoid giving the aerial alarm calls in the middle of the winter and at the end of the winter season. Finally, we expected that both dominant males and subordinate males would warn willow tits (*Poecile montanus*), heterospecific group members present during the glider presentations irrespective of the season. This is because heterospecific individuals provide the same basic anti-predator benefits at a significantly reduced competition cost, which does not affect the chance of juvenile crested tits becoming territory owners.

## 2. Methods

### (a) Study site, birds and dominance hierarchies

Experimental trials were performed between September and March of 1999**–**2003, 2004**–**2005, 2006**–**2007, 2010**–**2011, 2013**–**2014, 2016**–**2019, 2020**–**2021 in a 75- to 105-year-old coniferous forest dominated by Scots pine *Pinus sylvestris* and Norway spruce *Picea abies* near the town of Krāslava (55°87′ N, 27°19′ E) in southeastern Latvia [22].

We studied 25 mixed-species groups consisting of 104 (mean number of individuals = 4.16, s.d. = 0.34) crested tits as permanent group members. The mixed-species groups also contained willow tits (mean number of individuals = 4.08, s.d. = 0.28), coal tits (*Periparus ater*) (mean number of individuals = 5.0, s.d. = 0.43), great tits (*Parus major*) (mean number of individuals = 0.88, s.d. = 0.33), nuthatches (*Sitta europea*) (mean number of individuals = 2.12, s.d. = 0.33), and goldcrests (*Regulus regulus*) (mean number of individuals = 4.52, s.d. = 0.87). Crested and willow tits were marked with individually recognizable plastic rings and were aged and sexed. Only groups with all crested and willow tit individuals properly sexed and aged (as adult or juvenile; for more detail see [5]) were included in the analyses. To maintain independence of effects, we never sampled the same groups in consecutive wintering seasons, but we did retest groups living on the same territories.

Dominance order was measured within each group by observing pairwise interactions between birds at temporary feeders filled with sunflower seeds and fat. The food was provided during observation hours only and the birds were habituated to come to the feeders in the territories when hearing a specific auditory signal. To determine individual rank, we followed the procedures of Koivula & Orell [4] and Krams *et al*. [5]. The dominant won more interactions than the subordinate within each dyad (two-tailed sign-test, *p* < 0.001).

### (b) Social contexts and alarm call production

To elicit aerial alarm calls (figure 1), we simulated predation threat by flying a model of a male sparrowhawk (*Accipiter nisus*) above the feeder just when the focal individual excavated sunflower seeds out of solidified fat. We used a cardboard model of the predator scaled 1 : 18 of the natural size of the male sparrowhawk. The model was released along a fishing line from a distance of *ca* 10 m. The model's simulated speed was around 8–10 m s^−1^, corresponding to the speed of a slowly gliding sparrowhawk. The total length of the fishing line was 6 m. The predator passed the feeder 3.5 m above the head of the focal bird. At the moment of the predator's release, it was seen only by the focal individual feeding at the feeder. To avoid a focal bird's overexposure to the stimuli, we conducted a maximum of one trial per group per day. We conducted the trials during the following three seasons: at the beginning of the wintering season (September to the beginning of October), the middle of the winter (December) and the end of the wintering season (March). During each period (the beginning of the winter, the middle of the winter, the end of the winter), we simulated the presence of the predator for adult male crested tit and juvenile male crested tit 2–6 times for each of the following three social contexts: one focal individual foraging with another focal individual, the focal individual foraging together with one or two willow tits and the focal bird foraging alone. We created the situations when focal birds attended the feeders alone or accompanied by only a specific group mate by capturing females and adult or juvenile males in baited traps covered by fabric at one place and attracting the remaining birds by acoustic signals to the feeders in other parts of the territory. In some cases, focal birds arrived alone at the feeders, and no manipulations of other group members were needed. During each trial, we observed whether the focal bird produced aerial alarm calls or kept silent.

### (c) Statistical analyses

A binary logistic generalized linear mixed-effects model (GLMM) as implemented in software R 4.1.1. [23] package lme4 [24] was used to assess the influence of bird age, social context (with three levels: alone, with heterospecific willow tits, with another conspecific male), the time of the season and all two-way interactions between these factors on the probability of the production of aerial alarm calls. As there were multiple observations for each bird, bird identity was used as the random factor in the model to account for repeated measures. If the interaction term between the factors was significant, we performed *post hoc* comparisons of levels of one factor within levels of the second factor using a pairwise Tukey-adjusted comparison of estimated marginal means from the model as implemented in the package emmeans [25].

## 3. Results

Crested tits gave aerial alarm calls in 907 trials. They gave a series of three consecutive aerial alarm calls in two trials, two alarm calls during six trials and one aerial alarm call during the rest of the 899 trials. GLMM showed that all two-way interactions on the probability of giving alarm calls (figure 1) in the model were significant: bird age–social context (*χ*^2^ = 6.12, *p* = 0.047), bird age–season (*χ*^2^ = 8.85, *p* = 0.012) and social context–season (*χ*^2^ = 96.74, *p* < 0.0001).

**Figure 2.**
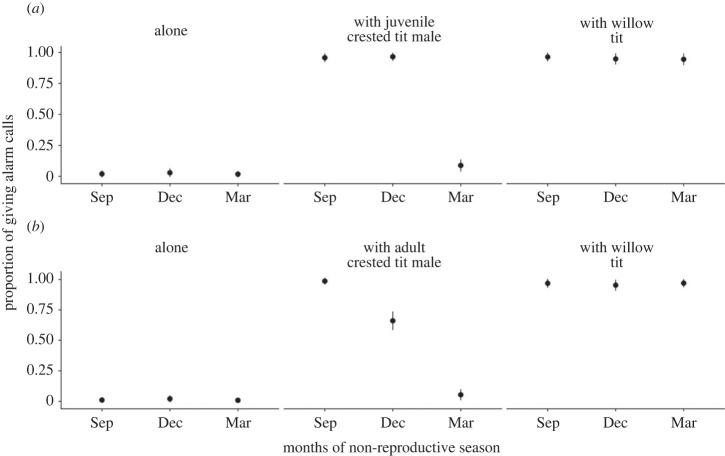
Production of aerial alarm calls by (*a*) adult male crested tits and (*b*) juvenile male crested tits across the non-reproductive season and social situations: detection of the predator when foraging alone, when foraging with other conspecific males and when foraging with heterospecific willow tits. The proportion of giving calls means the share of trials in which the focal individual produced aerial alarm calls.

Aerial alarm call production varied significantly between all the social contexts in the wintering season (all *p*-values < 0.05; figure 2*a,b*). A significant difference was seen between alone and with heterospecific willow tits at the beginning of wintering season in September (*z*-ratio −10.12, *p* < 0.0001; figure 2*a,b*) and at the end of the season in March (*z*-ratio −10.06, *p* < 0.0001; figure 2*a,b*). The probability of giving aerial alarm calls significantly differed between social contexts when feeding alone and when foraging with another conspecific male in September (*z*-ratio −10.54, *p* < 0.0001), but there were no differences in March (*z*-ratio −2.08, *p* = 0.094; figure 2*a,b*).

When foraging with heterospecific willow tits and foraging alone, there were no significant differences between the time of the season (all *p*-values < 0.005; figure 2*a,b*). When juveniles and adults were feeding together with each other at the end of the wintering season, there were no significant differences in the probability of giving alarm calls between the two social contexts (all *p*-values > 0.05; figure 2*a,b*) and also between these social contexts and when feeding alone (all *p*-values > 0.05; figure 2*a,b*).

Adult males and juvenile males showed significant differences between all social contexts within the age group (all *p*-values < 0.005; figure 2*a,b*), but within social context there was a significant difference only between adult and juvenile males when another conspecific male was present (*z*-ratio 2.89, *p* = 0.004; figure 2*a,b*).

Adult males showed significantly greater aerial alarm call production in September and December compared to March (*p*-values < 0.0001 and 0.0002; figure 2*a*), but juvenile males had the highest alarm call production in September compared to December and March (*p*-values 0.0001 and 0.0099; figure 2*b*). The probability of alarm call production was significantly higher in adult males than juvenile males in December (*z*-ratio 3.289, *p* = 0.001; figure 2*a,b*). However, there were no differences between age groups in September and March (*p*-values 0.853 and 0.482; figure 2*a,b*).

## 4. Discussion

Accessing information shared by members of social units is a crucial advantage of group living, but provision of key signals may be species, sex or age dependent. We determined that juvenile male crested tits begin withholding predator detection information (and shared vigilance) towards dominant male adults significantly earlier in the winter than dominant males begin withholding the same from juvenile males. Our work potentially demonstrates a subtle age and sex-specific behaviour associated with differences in the long- and short-term needs of social group members. Individual-specific constraints and priorities manifest in different types of cooperative, selfish and altruistic interactions among social group participants [19,20]. Birds in the family Paridae exhibit extreme social, vocal and cognitive complexity [26] and are more than capable of strategic withholding of alarm calling [2]. In these groups, male crested tits are non-kin and the decline of benefit from shared vigilance coincides with an increase in mate, food and territory competition that is not tempered by kin selection. We suggest that for juvenile males, a lack of territory ownership increasingly constrains their willingness to protect unrelated dominant males from harm as winter comes to an end [7]. For the dominant male, withdrawal of shared vigilance with subordinate males begins later in the winter, coinciding with the onset of breeding and the need to expel non-mate conspecifics from the territory.

Other possible explanations for the variation in juvenile alarm calling are not convincing. For example, mid-winter may be so stressful for inexperienced male crested tits, whose foraging may be clumsy, that they cannot afford to call while searching for limited foods (but this does not account for their willingness to call when willow tits are present). Further, it is possible that adult male crested tits do not respond to the alarms of juveniles, and once juvenile male crested tits learn this (during early winter), they stop warning the adults by mid-winter. If adult male crested tits perceived the juveniles to be too inexperienced to be reliable anti-predator signallers (e.g. [27]) and ignored them, juveniles may stop calling in mid-winter simply because they get no response (and their cost therefore outweigh benefits given). In this case, willow tits would need to be unaware of the juvenile crested tit males' unreliability to explain why they receive alarms, and this is unlikely given their comparable social and cognitive complexity to crested tits. We conclude that the most parsimonious and best-supported interpretation of selective alarm calling behaviour of juvenile crested tit males is selfishness as explained above. This interpretation aligns well with current understanding of how costs and benefits of sociality vary (in this case, temporally) among conspecific social group members.

Despite their differences, dominant and subordinate males continued to warn each other with aerial alarm calls at the end of winter, though very infrequently. These few warnings remaining at winter's end could be owing to previous familiarity [28] and reciprocal altruism as detected in great tit wintering in non-territorial groups [29]. Presumably, warning permanent group members may help maintain the group size constant, improving predator detection [7,30]. Social groups of crested tits are dominance-structured, suggesting that dominants enjoy more benefits from being social and survive better than subordinate individuals. Aggressive behaviour of adult crested tit males may make wintering in groups less attractive to subordinate individuals; Hogstad [31] showed that dominant aggression often temporarily causes subordinates to leave the groups. However, the detection of an approaching predator may be significantly reduced in smaller groups [30,32], suggesting an association between the aggressive behaviour of dominant individuals and the risk of being attacked by predators. Therefore, warning their subordinate group mates may be how dominant individuals compensate for their aggressive behaviour [5].

Importantly, adults warned their subordinate group mates at high frequency in the middle of the winter, even though the subordinate males were giving 50% fewer warnings. Given that this would improve the survival of unrelated subordinates through the worst periods of winter, it seems disadvantageous on the part of adult males. An altruistic act of helping a non-relative only pays the altruist if it is directed at a particular individual who reciprocates on a later occasion [33]. However, adult males become very familiar with their permanent flock mates over weeks and months. It could be that continued warning of subordinate group mates that do not warn others might be based on dominant males' attempts to maintain their groups' stability during continued harsh conditions. Even though reciprocation by subordinates is declining, benefits accruing to the dominant remain higher with subordinate males in the groups than if the subordinates left too soon.

Juvenile male crested tits decreased the frequency of their alarm calling behaviour in mid-winter. Although the high-pitched aerial alarm calls can be heard and often located by predators, the observed change in alarm calling of juvenile crested tits was supposedly not caused by predators. Given that subordinate males did not withhold alarms when other birds were present, but only when the dominant male was nearby, we assumed that this behaviour functions to increase mortality risk only to the dominant male. This would enhance the chances that the subordinate male could rise in the dominance hierarchy and acquire a territory for breeding in time to secure spring breeding opportunity. Thus, this study shows that subordinates are not just social satellites accepted by dominant territory owners. However, they can actively affect and manipulate the survival prospects of their dominant group mates. Future research is needed to show whether juvenile males that avoid warning adult males have more chances to breed in their winter territories than juvenile males who continue warning dominant males until the end of the non-reproductive season.

In contrast with what has been observed regarding alarm calling behaviour between adult and juvenile male crested tits, dominant and subordinate males warned their heterospecific group mates independent of the phase of the wintering season, suggesting little competition between the species and significant anti-predator benefits of heterospecific flocking. Interspecific competition plays little or no role in complementary mixed-species groups where heterospecific group members/social partners obtain different complementary benefits [1]. It has been found that mixed-species associations between parids and other ecologically related birds remain important outside the non-breeding season. These groups are based on positive ecological relationships and interspecific information exchange [34–37]. This suggests that more research needs to be done on warning heterospecific group members during the reproductive season.

In this study, we could not discriminate between selfishness in not warning a social partner and spite in social relationships [20]. For example, juvenile males might be less willing to alert the dominating males because of the aggressive attacks by the dominants. However, antagonistic interactions between group members can be seen throughout the winter season. Therefore, it is not likely that the forthcoming spring season will explain the rise of spiteful behaviour of subordinate crested tit males.

Finally, it would be important to compare the present results with the field observations of parid responses towards real predators under different social contexts. This would improve our understanding of the evolution of bird aerial alarm calls and provide recommendations for researchers to compare the use of artificial predator models and natural predators.

## Data Availability

Data file is available from the Zenodo repository: https://doi.org/10.5281/zenodo.7634270 [38].
